# Recruitment into antibody prevalence studies: a randomized trial of postcards vs. letters as invitations

**DOI:** 10.1186/s12874-023-01992-8

**Published:** 2023-07-22

**Authors:** Catherine Ley, Heying Duan, Julie Parsonnet

**Affiliations:** 1 Division of Infectious Diseases and Geographic Medicine, Department of Medicine, Stanford University, 300 Pasteur Drive, Stanford, CA 94305 USA; 2 Division of Nuclear Medicine and Molecular Imaging, Department of Radiology, Stanford University, Stanford, USA; 3 Department of Epidemiology & Population Health, Stanford University, Stanford, USA

**Keywords:** Recruitment, Postal mailing, Seroprevalence survey, Representative sample, Probability sampling

## Abstract

**Background:**

In a potential epidemic of an emerging infection, representative population-based serologic studies are required to determine the extent of immunity to the infectious agent, either from natural infection or vaccination. Recruitment strategies need to optimize response rates.

**Methods:**

Within a seroepidemiologic study to determine the true burden of SARS-CoV2 infection in two Bay Area counties, we evaluated whether letter (L) or postcard (P) invitations with reminders were more effective at recruiting participant households. Using geographic, probability-based sampling, 9,999 representative addresses, split between Santa Clara and Solano counties, were randomized to receive an initial invitation (L or P); a randomized reminder mailing sent two weeks later to all non-respondents created four mailing type groups (L/L, L/P, P/L, P/P). Interested households provided contact information via survey to perform blood spot collection at home for testing and then receive SARS-CoV2 serology results. Comparison of demographics among respondents and non-respondents used census tract data.

**Results:**

Receiving any reminder mailing increased household response rates from 4.2% to between 8–13% depending on mailing combination. Response rates from two letters were 71% higher than from two postcards (13.2% vs. 7.7%, OR = 1.83 [95% CI: 1.5–2.2]). Respondents were older, more educated and more likely white than non-respondents. Compared to Solano county, Santa Clara county had different demographics and increased household response rates (L/L: 15.7% vs 10.7%; P/P: 9.2% vs. 6.1%; *p* < 0.0001); the effect of mailing types, however, was the same (L/L vs. P/P: Santa Clara: OR = 1.83 [95% CI: 1.4–2.3]; Solano: OR = 1.84 [95% CI:1.4–2.5]).

**Conclusion:**

Letters, as both invitations and reminders, are a more effective recruitment tool than postcards and should be considered when seeking a representative population-based sample for serological testing.

**Supplementary Information:**

The online version contains supplementary material available at 10.1186/s12874-023-01992-8.

## Introduction

Reported cases of a disease with epidemic potential can represent a small, biased fraction of all infections, as illustrated by the current pandemic of COVID-19 and SARS-CoV2 [1]. In the context of a possible epidemic, where public health response and planning, policy decisions and pandemic preparedness strategies are required, population-based, longitudinal serologic studies are necessary, both to determine the extent of immunity – either naturally acquired or from vaccination—and to evaluate the pace of infection and immunity acquisition [2, 3].

Representative samples of populations are best identified via physical mailing addresses; in the US, address-based samples (ABSs) generated from the US Postal Service Delivery Sequence File—which is a list of all addresses to which mail is delivered by the USPS—are based on nearly all US households (90–98%) [4]. Address-based recruitment strategies today are often mixed-mode, where a mailed invitation precedes a second web-based or telephone survey; initial invitations have included letters, perceived as likely to contain important information, and postcards, perceived as easily accessible and less expensive than letters [5]. In contrast, direct recruitment by social and other web-based media tends to be biased as access to the internet remains lower among Hispanic and African American households, among older adults, and among rural, low-income and less educated households [6, 7]; additional sources of non-representative samples from web-based media include both the self-selection and the non-response rate of potential participants, among others [8, 9].

Over the first two years of the COVID-19 pandemic, the public was aware of and generally interested in SARS-CoV2, and, given that testing kits were essentially unavailable at that time [10, 11], enthusiasm for at-home testing was high [12]. We implemented a series of seroepidemiologic studies to determine the true burden of SARS-CoV2 infection in two Bay Area counties in California, and anticipated that response rates to an unsolicited invitation to participate in serologic testing for SARS-CoV2 would likely have a better response than that typical for unsolicited mailings, purported to range from 2.7% to 4.4% [13].

To recruit participants, we sent an invitation to a population-based sample of households. The invitation stated that up to three members of the household could receive free serologic testing for SARS-CoV2 by collecting a finger stick blood spot at their home, completing a questionnaire and returning the blood spot card by mail; the recipient accepted participation either online or by telephone. With the goal of enhancing recruitment, we assessed whether type of mailing—postcard or letter—affected response rates. For our first serologic study, we contacted a random selection of 10,000 households in both Santa Clara and Solano counties and evaluated differential response rates to postcards and letters.

## Material and methods

CA-FACTS, the Californians Fighting Against Coronavirus Together Study, was a collaboration between Stanford University (Stanford, CA) and the public health departments of Santa Clara and Solano counties (San Jose and Vallejo, CA, respectively). It sought to determine the prevalence across time of SARS-CoV2 antibodies in the population using a highly sensitive and specific serologic test developed at Stanford University. Recruitment material for CA-FACTS was developed in four languages for Santa Clara county (English, Spanish, Vietnamese, Mandarin Chinese) and in two for Solano county (English, Spanish) at Stanford University and then designed and printed in both a postcard and a letter format (Gunderson Direct Inc., Hayward, CA) (Supplemental Information). The postcard was by necessity quite brief but appeared enthusiastic, describing the household as having been “chosen;”; the letter was more detailed and appeared official, describing the household as having been “selected at random” (Supplemental Information: Recruitment invitation). The overall cost of printing and mailing was $0.15 for a postcard and $0.30 for a letter.

**Fig. 1:**
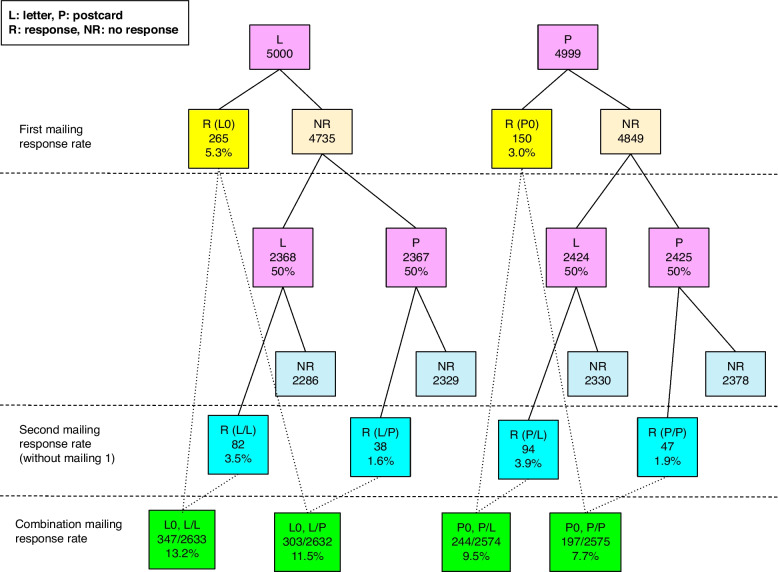
**Number and percent of individual households assigned to either letter or postcard invitations at each of the two mailings.** Pink indicates randomization to a letter (L) or a postcard (P); numbers of responses (R) and response rates are bright yellow for the first mailing, bright blue for the second mailing and bright green for the combination of mailing types; numbers of non-respondents (NR) are in pale yellow for the first mailing and pale blue for the second mailing. Denominators for the combination mailing rates include those who responded to the first mailing in addition to those assigned to the category for the second mailing. (Note that in this figure the second mailing rate does not include the initial responses.)

To test the recruitment material format, we obtained a list of 10,000 representative addresses, half each in Santa Clara and Solano counties, using geographic, probability-based sampling (Marketing Systems Group, Horsham, PA). To determine whether the response rate to a postcard was the same as to a letter, addresses were randomized to receive either the postcard or the letter in an initial mailing (Fig. 1). A reminder mailing was then sent to all non-respondents, approximately two weeks after the initial mailing. To determine whether the response to this second mailing was simply a reminder of the initial invitation, or instead was associated with mailing type, we again randomized the type of mailing sent. If the initial mailing had been a postcard, then, randomly, one half of non-respondents received a postcard and one half a letter; if the initial mailing had been a letter, then, randomly, one half received a letter and one half received a postcard. The reminder postcards and letters were identical to the initial postcards and letters.

Responses to the invitation once received in the mail were made using a short web-based entry form (Gauss, Menlo Park, CA) or by brief telephone survey via interactive voice response (Twilio.com) with all data collected and managed using REDCap hosted at Stanford University [14].

Characteristics of the two counties were identified from census data [15]. To identify potential demographic differences both between respondents and non-respondents and between respondents to different combinations of mailings, we identified specific characteristics for each census tract from the American Community Survey 1 year estimates from 2020, including the proportions of the population that: was aged 65 years or greater, had a college degree or more, identified as of Hispanic ethnicity and identified either as Black/African American only, Asian only or White only [16].

Comparisons of household response rates to both the initial and the second mailings (mailing #1, mailing #2) and times to response, overall and by county, were performed using the Chi-square test or Wilcoxon signed-rank test, as appropriate. Odds ratios (OR) and associated 95% confidence intervals (CI) compared combinations of mailing types (responded to the initial postcard *or* to the initial postcard with a reminder postcard (P0/PP); responded to the initial postcard *or* to the initial postcard with a reminder letter (P0/PL); responded to the initial letter *or* to the initial letter with a reminder postcard (L0/LP); and, responded to the initial letter *or* to the initial letter with a reminder letter (L0/LL)). Logistic regression models were performed to compare census tract characteristics of respondents and non-respondents, and included the different mailing combinations of postcards only (P0/PP) and letters only (L0/LL). Additional models among respondents only compared characteristics of census tracts and of households (language spoken, number of people tested by household) by mailing type. *P*-values of < 0.05 were considered significant. All analyses were conducted in SAS V9.4 (SAS Inc., Cary NC).

## Results

A total of 9,999 invitations to participate in CA-FACTS were sent to addresses in Santa Clara and Solano counties in the initial mailing in October, 2020 (Fig. 1, Table 1). Of these, 4,999 (50%) were postcards and 5,000 were letters. For this first mailing, a significantly higher household response rate was seen for the letter invitation than for the postcard invitation (Table 1). Similar results were seen for the second mailing approximately two weeks later: a total of 9,584 reminder invitations were mailed, consisting of 4,792 (50%) letters and 4,792 postcards. Again, the household response rate for the reminder letter was significantly higher than for the reminder postcard. The median time from sending the invitation to receiving a response, either as a first or a second mailing, was not statistically significantly different between the letter and the postcard.

**Table 1:** Comparison of invitation response rates and days to response for first and second mailings and their combinations by mailing type (postcard and letter)

		**Mailings sent**	**Responded**	**No response**	**Response rate (%)**	***P* 1**	**OR**	**CI**	**Days to response **^**a**^	***P* 2**
		N	N	N	%				Median (Q1-Q3), range	
Mailing #1	P	4999	150	4849	3.00	< 0.001	1		5 (3–7), 2–35	0.07
	L	5000	265	4735	5.30		1.81	1.48–2.22	4 (3–7), 2–31	
	Total	9999	415	9584	4.15					
Mailing #2 ^b^ (reminder)	P	4942	235	4707	4.76	< 0.001	1		6 (3–15), 1–45 ^c^	0.65
	L	5057	441	4616	8.72		1.91	1.63–2.25	5 (3–12), 1–49 ^c^	
	Total	9999	676	9323	6.76					
Combinations of mailings ^b^	P0 or P/P	2575	197	2378	7.7	< 0.001	1		5 (3–9), 1–45	0.87
	P0 or P/L	2574	244	2330	9.5		1.26	1.04–1.54	5 (3–9), 1–49	
	L0 or L/P	2632	303	2329	11.5		1.57	1.30–1.90	5 (3–7), 1–33	
	L0 or L/L	2633	347	2286	13.2		1.83	1.52–2.20	5 (3–8), 1–49	

*P0* Postcard only, *P/P* Postcard/postcard, *P/L* Postcard/letter, *L0* Letter only, *L/P* Letter/postcard, *L/L* Letter/letter. *P1*: comparison of response rates, *P2*: comparison of days to response, *OR* Odds ratio, *CI* 95% confidence interval, *Q1-Q3* Interquartile range

^a^ Time to response: for those missing survey completion date (~ 20%), estimated as that of next dated record in database

^b^ Households who responded to their first mailing are included in each category, as they would have received a second mailing had they not already responded (alternatively, N received after second mailing but not including initial mailing]: P: 85, L: 176)

^c^ Calculation includes only the responses from the second mailing

Sending a reminder after an initial invitation increased the response rate, with the combination of mailing, both invitation and reminder, being a predictor of response (< 0.001). Households who received two letters were 1.83 times more likely to respond compared to those who received two postcards (13.2% vs. 7.7%); those who received an initial letter invitation with a postcard reminder, or an initial postcard invitation with a letter reminder were 1.6 and 1.3 times more likely to respond than those who received two postcards (11.5 and 9.5% vs. 7.7%, respectively). Median time to response was not different among the different combinations of mailing types, with a median of five days, although the range of time to response was very wide.

The two counties have different population sizes (1,885,508 for Santa Clara and 451,716 for Solano), with statistically significant differences in education levels, age distribution, ethnicity and racial composition (all *p* < 0.0001) (Supplemental Table S1). Santa Clara county tended to have higher response rates then Solano county (two postcards: 9.2% vs. 6.1%; two letters: 15.7% vs. 10.7%; *p* < 0.0001); the effect on response by mailing type, however, was similar between counties, with a response from two letters 1.8 times more likely than from two postcards for both Santa Clara and Solano counties (Supplemental Table S2).

Comparison of respondent and non-respondent households overall and by county using census tract data showed that respondents came from census tracts where residents were likely to be more educated (proportion with a college degree or more), have older median age (proportion aged 65 years or more), not identify as Hispanic, and were either slightly less likely to be Asian (Solano only) or Black (Supplemental Table S1). Multivariable modeling that maintained mailing combinations P0/PP and L0/LL in all models identified few consistent predictors of response by county other than mailing type and increased education due to significant collinearity of the other census characteristics (for example, census tracts with a high proportion of White only respondents had a lower proportion of Black only or Hispanic residents, and more older residents). All models showed that respondents were approximately twice as likely to have received letter invitations than postcard invitations. Models among respondents only that examined predictors of response to letters compared to response to postcards did not identify any consistent predictors either overall or by county (data not shown).

## Discussion

In this study, we evaluated the response rate to either postcard or letter invitations and reminders to participate in CA-FACTS, a population-based seroepidemiologic study to determine the burden of SARS-CoV2 infection in two Bay Area Counties in California. Our household response rates with two mailings, an initial one followed by a reminder two weeks later, was dependent on mailing type combinations: households receiving both a letter invitation and a letter reminder were 71% more likely than those receiving both a postcard invitation and reminder to enroll in the study – 13% vs. 8%—making two letter invitations a more effective recruitment tool than two postcards. This effect of mailing type on response rate was the same for both Santa Clara and Solano counties despite their very different population characteristics and response rates, confirming that letters were more appealing than postcards across the board.

Reminders are important: paper invitations with paper reminders have been shown to improve response rates over a single mailing [17]. Indeed, in this study, use of any type of reminder mailing improved the household response rate, which was already on the high end for a typical “cold” mailing [13], from 4.2% to 10.5%. External events played a large part in overall interest in participating in our study: during October 2020, Santa Clara county moved into a lower COVID-19 risk category (the “orange tier” within California’s risk framework), allowing resumption of indoor dining and gatherings [18], while Solano county remained in the higher red tier [19]. By the middle of November, however, the Delta variant was surging and both counties were in the highest risk category, the purple tier, which included severe restrictions on usual activities. COVID-19 was omnipresent in the news, at-home testing kits were still on the horizon, and vaccination was far off, so the incentive to participate—learning the serostatus of up to three people in the household—was high.

Our finding updates two large studies: one of almost 14,000 households from 2005 showing that the completion rate of a telephone survey with advance letters was greater than with advance postcards, and another of 17,808 households from 2022 showing that the completion rate of a web-based survey was greater with advance letters with or without incentives than with postcards with or without incentives; additionally, both studies showed that letters were more cost-effective than postcards [20, 21]. Although postcards are brief, readily accessible without needing to open an envelope, and usually have costs roughly half that of a letter (including printing and mailing), letters appear more formal and may be perceived to carry important or interesting information to which people may be more likely to respond, especially when the sender is clearly identified as a non-commercial, well-respected entity, such as, in our study, Solano and Santa Clara counties’ public health departments and Stanford University [5, 22]. Letters additionally are thought to have greater long-term memory effects than postcards, as the envelope must be opened, the letter extracted and read, and then either stored or discarded. Differential recall of receiving the invitation possibly had a role in this study [5], given that all combinations of mailings that included at least one letter had higher response rates than that with only postcards.

Despite the positive effect of letters, we wish to emphasize that two postcards, one initial and one reminder, can be used as an effective recruitment tool, as their use more than doubled the response rate over an initial postcard alone. It is important to note, however, that in some cases postcards should not be used for recruitment, such as when the invitation reveals an element of health status, or when the sample is identified from a patient population frame [23]. Clearly, the selection of effective recruitment tools remains dependent on the research study under consideration.

Limitations to our study include the biased respondents despite our representative sample, as respondents were more likely to be well educated, older and more White than present in the counties’ populations overall. This response bias is well known especially for racial groups [24, 25] – and subsequent mailings for our serosurvey oversampled Hispanic and Black populations. The increased level of concern about COVID19 in the elderly and the more educated likely played a part as well, as has been shown by the rapid and high vaccination uptake in these groups [26]. Census tract demographics were not able to identify differences between respondents and non-respondents other than a higher proportion of college educated residents or White only residents; we did not identify any census-tract or household level demographic characteristics among respondents that linked to mailing type.

In summary, letter invitations with letter reminders are considerably more effective than postcards in recruiting population-based samples and should be used as essential seroepidemiologic studies are implemented to understand population immunity against epidemic agents and to prevent or decrease infection.

## Supplementary Information

**Additional file 1:** **Supplemental Table S1.** Census-based characteristics of each county (percent), and of census tracts (median percent, interquartile range) among respondents and non-respondents, overall and by county. **Supplemental Table S2.** Comparison of invitation response rates for first and second mailings and their combinations by mailing type (postcard and letter), by county. **Supplemental information.** Recruitment invitation for Santa Clara County.

## Data Availability

All data generated or analyzed during this study are included in this published article and its supplementary information files.

## References

[1] Murray CJL (2022). COVID-19 will continue but the end of the pandemic is near. Lancet.

[2] WHO https://www.who.int/news/item/03-02-2022-true-extent-of-sars-cov-2-infection-through-seroprevalence-studies.

[3] Winter AK, Hegde ST (2020). The important role of serology for COVID-19 control. Lancet Infect Dis.

[4] AAPOR https://www.aapor.org/Education-Resources/Reports/Address-based-Sampling.aspx#SECTION%208.

[5] Dillman DA (2000). Mail and internet surveys: the tailored design method.

[6] Public Policy Institute https://www.ppic.org/publication/californias-digital-divide/.

[7] Hsia J, Zhao G, Town M (2020). Estimating Undercoverage Bias of Internet Users. Prev Chronic Dis.

[8] Bennetts SK, Hokke S, Crawford S (2019). Using paid and free Facebook methods to recruit Australian parents to an online survey: an evaluation. J Med Internet Res.

[9] Whitaker C, Stevelink S, Fear N (2017). The use of facebook in recruiting participants for health research purposes: a systematic review. J Med Internet Res.

[10] KFF https://www.kff.org/report-section/rapid-home-tests-for-covid-19-issues-with-availability-and-access-in-the-u-s-issue-brief/.

[11] ASM https://asm.org/Press-Releases/2020/October/Shortages-of-COVID-19-and-Other-Testing-Supplies-I.

[12] JAMA Network https://jamanetwork.com/journals/jama/fullarticle/2772299.

[13] ReportLinker https://www.repo4rtlinker.com/p04442212/Direct-Mail-Advertising-Global-Market-Briefing.html?utm_source=GNW.

[14] Harris PA, Taylor R, Thielke R, Payne J, Gonzalez N, Conde JG (2009). Research electronic data capture (REDCap)—a metadata-driven methodology and workflow process for providing translational research informatics support. J Biomed Inform.

[15] Census.gov https://www.census.gov/quickfacts/fact/table/solanocountycalifornia,santaclaracountycalifornia,CA,US/HSG860220; Accessed 9/2/2022.

[16] American Community Survey https://www.census.gov/programs-surveys/acs.

[17] Sakshaug JW, Vicari B, Couper MP (2019). Paper, e-mail, or both? Effects of contact mode on participation in a Web survey of establishments. Soc Sci Comput Rev.

[18] Sccgov.org https://covid19.sccgov.org/news-releases/pr-10-13-2020-scc-moves-to-ca-state-orange-tier.

[19] Solanocounty.com https://www.solanocounty.com/depts/ph/coronavirus_links/coronavirus_press_releases_and_information.asp.

[20] Hembroff LA, Rusz D, Rafferty A, Mcgee H, Ehrlich N (2005). The cost-effectiveness of alternative advance mailings in a telephone survey. Public Opin Q.

[21] Brenner PS, Buskirk TD (2022). Scratch the scratch-off: testing prepaid and conditional incentives with postcard and letter invitations in a web-push design with an address-based sample. Field Methods.

[22] Edwards PJ, Roberts I, Clarke MJ (2009). Methods to increase response to postal and electronic questionnaires. Cochrane Database Syst Rev.

[23] Beskow LM, Botkin JR, Daly M (2004). Ethical issues in identifying and recruiting participants for familial genetic research. Am J Med Genet A.

[24] Compton J, Glass N, Fowler T (2019). Evidence of selection bias and non-response bias in patient satisfaction surveys. Iowa Orthop J.

[25] Simsek I, Manemann SM, Yost KJ (2020). Participation bias in a survey of community patients with heart failure. Mayo Clin Proc.

[26] Yougov.com https://today.yougov.com/topics/politics/articles-reports/2021/04/19/college-graduates-report-higher-vaccination-rates.

