# Supplementary material for: Recruitment into antibody prevalence studies: a randomized trial of postcards vs. letters as invitations
Source: BMC Med Res Methodol. 2023 Jul 22;23:170. doi: 10.1186/s12874-023-01992-8 (PMC10363298; doi:10.1186/s12874-023-01992-8)

|  | By county ^a^  Percent | | By census tract  Median percent (interquartile range) | | | | | | | | |
| --- | --- | --- | --- | --- | --- | --- | --- | --- | --- | --- | --- |
|  |  | | Overall | | | Santa Clara County | | | Solano County | | |
|  | Santa Clara | Solano | Resp ^b^ | Non-Resp ^b^ | P ^c^ | Resp | Non-Resp | p | Resp | Non-Resp | p |
| Male | 51 | 52 | 50  (48-52) | 50  (48-52) | 0.18 | 50  (48-52) | 50  (48-52) | 0.58 | 49  (47-51) | 49  (47-51) | 0.76 |
| College or more ^d^ | 54 | 28 | 50  (34-72) | 35  (22 -54) | <0.0001 | 67  (55-78) | 56  (40-72) | <0.0001 | 30  (21-38) | 25  (17-35) | <0.0001 |
| Age 65+ years | 15 | 17 | 15  (11-20) | 14  (11-18) | <0.0001 | 14  (11-19) | 14  (10-17) | <0.0001 | 16  (12-22) | 15  (11-19) | 0.0002 |
| Hispanic | 25 | 29 | 18  (10-29) | 24  (16-34) | <0.0001 | 12  (7-23) | 21  (10-31) | <0.0001 | 26  (17-30) | .27  (18-35) | 0.0004 |
| White only | 51 | 59 | 41  (26-53) | 31  (19-44) | <0.0001 | 39  (26-50) | 31  (16-43) | <0.0001 | 43  (27-56) | 31  (20-46) | <0.001 |
| Black only | 3 | 15 | 2  (1-7) | 4  (2-15) | <0.0001 | 2  (1-2) | 2  (1-3) | <0.0001 | 0.8  (6-17) | 14  (7-18) | <0.001 |
| Asian only | 41 | 17 | 24  (11-38) | 21  (11-36) | <0.02 | 34  (24-49) | 35  (23-51) | 0.91 | 18  (6-25) | 12  (8-21) | 0.0002 |

**Supplemental Table S1:** **Census-based characteristics of each county (percent), and of census tracts (median percent, interquartile range) among respondents and non-respondents, overall and by county**

a: Overall population estimate as reported by census.gov (accessed 30Jan2023)

b: Res: respondents; Non-Resp: non-respondents. A total of 99 (17.2%) respondents and 1283 (15.2%) non-respondents were missing census tract information (p=0.29), 13.8% of the total households initially sent an invitation.

c: Wilcoxon rank sum test

d: Education level assessed in people over age 25 years.

**Supplemental Table S2: Comparison of invitation response rates for first and second mailings and their combinations by mailing type (postcard and letter), by county**

|  |  | **Santa Clara** | | | | | **Solano** | | | | |
| --- | --- | --- | --- | --- | --- | --- | --- | --- | --- | --- | --- |
|  |  | **Sent** | **Resp** | **No resp** | **Resp rate** | **OR [95%CI]** | **Sent** | **Resp** | **No resp** | **Resp rate** | **OR [95%CI]** |
|  |  | N | N | N | % |  | N | N | N | % |  |
| Mailing #1 | P | 2500 | 88 | 2412 | 3.52 | 1 | 2499 | 62 | 2437 | 2.48 |  |
|  | L | 2501 | 154 | 2347 | 6.16 | 1.80 (1.4-2.4) | 2499 | 111 | 2388 | 4.44 | 1.83 (1.3-2.5) |
|  | Total | 5001 | 242 | 4759 | 4.84 |  | 4998 | 173 | 4825 | 3.46 |  |
| Mailing #2  (reminder) ^a^ | P | 2467 | 143 | 2324 | 5.79 | 1 | 2475 | 92 | 2383 | 3.72 | 1 |
|  | L | 2534 | 265 | 2269 | 10.46 | 2.00 (1.5-2.4) | 2523 | 176 | 2347 | 6.98 | 1.92 (1.5-2.5) |
|  | Total | 5001 | 408 | 4593 | 8.16 |  | 4998 | 268 | 4730 | 5.36 |  |
| Combinations of mailing type ^a^ | P0 or P/P | 1294 | 119 | 1175 | 9.20 | 1 | 1281 | 78 | 1203 | 6.09 | 1 |
|  | P0 or P/L | 1294 | 145 | 1206 | 11.21 | 1.25 (1.0-1.6) | 1280 | 99 | 1181 | 7.73 | 1.29 (1.0-1.8) |
|  | L0 or L/P | 1327 | 178 | 1149 | 13.41 | 1.53 (1.2-2.0) | 1305 | 125 | 1180 | 9.58 | 1.63 (1.2-2.2) |
|  | L0 or L/L | 1328 | 208 | 1120 | 15.66 | 1.83 (1.4-2.3) | 1305 | 139 | 1166 | 10.65 | 1.84 (1.4-2.5) |

OR: odds ratio; CI: confidence interval; Resp: response; No Resp: No response

Mailing type: P0: postcard only; P/P: postcard/postcard; P/L: postcard/letter; L0: letter only; L/P: letter/postcard; L/L: letter/letter

^a^ Households who responded to their first mailing are included in each category, as they would have received a second mailing had they not already responded (alternatively, N received after second mailing but not including initial mailing: Santa Clara: P:55, L:111; Solano: P:30, L:65).

**Supplemental information: Recruitment invitation for Santa Clara County.**

1. Letter


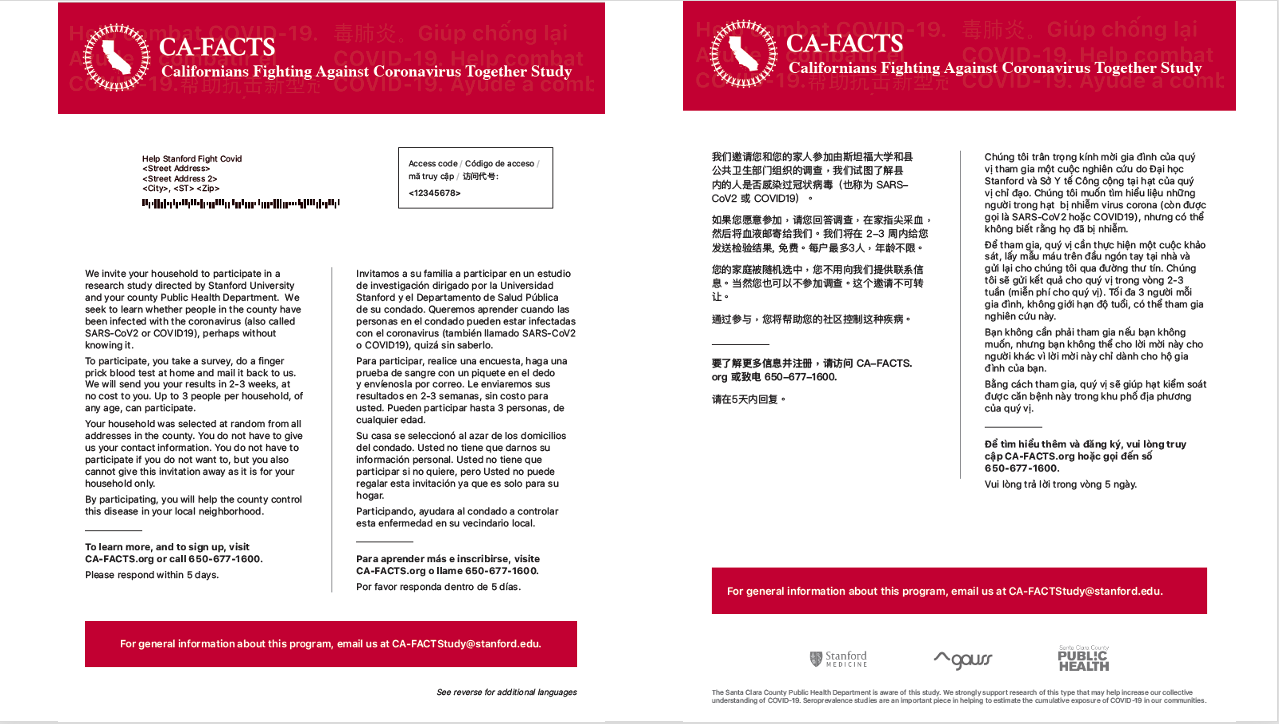


(B) Postcard


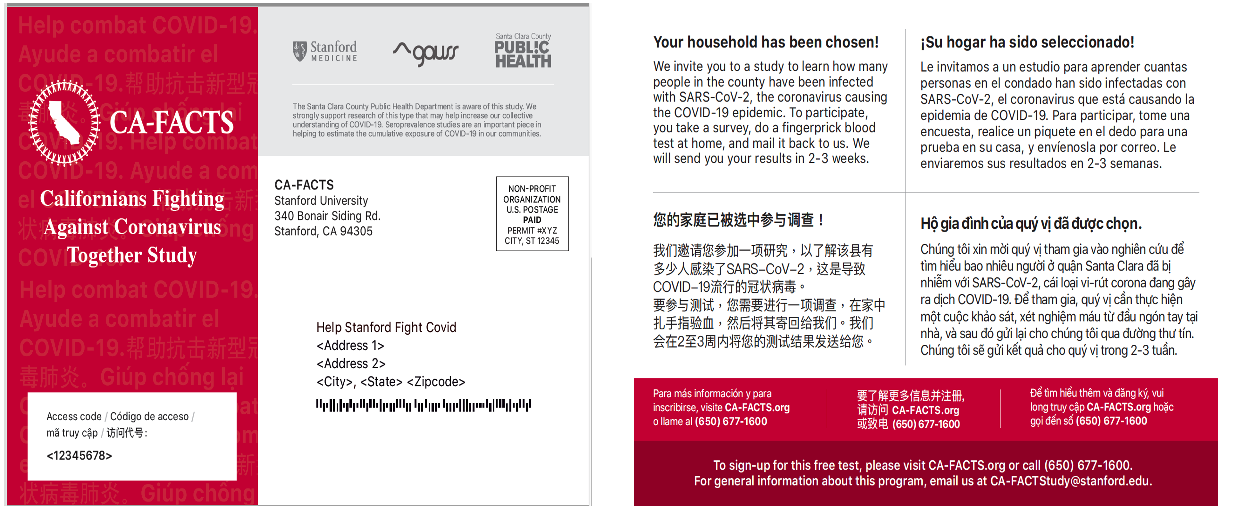

Supplement: Supplementary file 1 — Additional file 1: Supplemental Table S1. Census-based characteristics of each county (percent), and of census tracts (median percent, interquartile range) among respondents and non-respondents, overall and by county. Supplemental Table S2. Comparison of invitation response rates for first and second mailings and their combinations by mailing type (postcard and letter), by county. Supplemental information. Recruitment invitation for Santa Clara County. [file 12874_2023_1992_MOESM1_ESM.docx]
